# CMV Infection Attenuates the Disease Course in a Murine Model of Multiple Sclerosis

**DOI:** 10.1371/journal.pone.0032767

**Published:** 2012-02-29

**Authors:** Istvan Pirko, Rhonda Cardin, Yi Chen, Anne K. Lohrey, Diana M. Lindquist, R. Scott Dunn, Robert Zivadinov, Aaron J. Johnson

**Affiliations:** 1 Department of Neurology, Mayo Clinic, Rochester, Minnesota, United States of America; 2 Division of Infectious Diseases, Cincinnati Children's Hospital Medical Center, Cincinnati, Ohio, United States of America; 3 Department of Neurology and Immunology, Mayo Clinic, Rochester, Minnesota, United States of America; 4 Imaging Research Center, Cincinnati Children's Hospital Medical Center, Cincinnati, Ohio, United States of America; 5 Department of Neurology and Buffalo Neuroimaging Analysis Center, University of Buffalo, Buffalo, New York, United States of America; National Institutes of Health, United States of America

## Abstract

Recent evidence in multiple sclerosis (MS) suggests that active CMV infection may result in more benign clinical disease. The goal of this pilot study was to determine whether underlying murine CMV (MCMV) infection affects the course of the Theiler's murine encephalitis virus (TMEV) induced murine model of MS. A group of eight TMEV-infected mice were co-infected with MCMV at 2 weeks prior to TMEV infection while a second group of TMEV-infected mice received MCMV two weeks post TMEV. We also used 2 control groups, where at the above time points MCMV was replaced with PBS. Outcome measures included (1) monthly monitoring of disability via rotarod for 8 months; (2) in vivo MRI for brain atrophy studies and (3) FACS analysis of brain infiltrating lymphocytes at 8 months post TMEV infection. Co-infection with MCMV influenced the disease course in mice infected prior to TMEV infection. In this group, rotarod detectable motor performance was significantly improved starting 3 months post-infection and beyond (p≤0.024). In addition, their brain atrophy was close to 30% reduced at 8 months, but this was only present as a trend due to low power (p = 0.19). A significant reduction in the proportion of brain infiltrating CD3+ cells was detected in this group (p = 0.026), while the proportion of CD45+ Mac1+ cells significantly increased (p = 0.003). There was also a strong trend for a reduced proportion of CD4+ cells (p = 0.17) while CD8 and B220+ cell proportion did not change. These findings support an immunomodulatory effect of MCMV infection in this MS model. Future studies in this co-infection model will provide insight into mechanisms which modulate the development of demyelination and may be utilized for the development of novel therapeutic strategies.

## Introduction

Multiple sclerosis (MS) is the most common inflammatory demyelinating disease of the central nervous system (CNS). MS is the leading cause of non-traumatic disability among young adults in their most productive years [1]. Although the exact cause of MS remains elusive, it is widely accepted that the pathology of MS is mediated by the immune system in genetically susceptible hosts [2]. In addition to genetic risk factors, which determine the susceptibility and may influence disease severity, environmental factors are also suspected to contribute as disease initiating events [3]. Infections, especially of viral etiology, have long been suspected as environmental factors that may contribute to the development of MS in addition to environmental variables including vitamin-D deficiency [4]. Several viruses have been suspected as MS triggers. Currently, the most commonly studied virus which appears to be associated with MS is Epstein-Barr Virus (EBV), a member of the *Herpesviridae* family [5]. EBV establishes a persistent infection in B cells. Interestingly, EBV is also suspected to play a role in the pathogenesis of several classic autoimmune diseases, including polymyositis, SLE, anti-phospholipid antibody syndrome, rheumatoid arthritis, pemphigus vulgaris, giant cell arthritis, Wegener's granulomatosis, and polyarteritis nodosa [6]. A second herpes virus, human cytomegalovirus (HCMV), has also been proposed as a potential MS trigger [7]. However, more recent studies with extensive case ascertainment failed to demonstrate such an association [5]. Among systemic autoimmune conditions, elevated HCMV IgG titers were observed only in the sera of SLE patients [6].

In a recent study analyzing the role of active HCMV infection in MS cases, multiple analyses demonstrated a clear association between antibody positivity against HCMV and better clinical and MRI outcomes [8]. These analyses indicated that patients positive for antibodies against HCMV had significantly older age of disease onset, lower lifetime relapse rate, higher brain parenchymal fraction (BPF)on volumetric MRI, suggesting less brain atrophy. HCMV-positive patients who had higher antibody titer presented with lower T2 weighted lesion load and higher BPF compared to patients with lower levels. Of note, this doesn't mean that the antibody itself would be responsible for the protective effect; instead, it implies that recent active infection overall has a protective role, via a mechanism that can't be directly clarified in MS patients, but could be clarified via mechanistic studies in animal models of the above phenomenon. The above was the first study to suggest a protective role of HCMV infection in MS [8]. HCMV encodes multiple genes which serve to down modulate the immune response during infection. These immune suppressive aspects of HCMV infection could account for the protective effects observed in HCMV-infected MS patients [9], [10].

The goal of our study was to determine the extent murine CMV (MCMV) infection exerts a similar protective effect in a mouse model of MS. If protective, this model system could be analyzed further to identify potential therapeutic exploitations of the molecular mechanism(s) responsible for this effect. In the current study, we utilized the TMEV infection based model of MS [11]. Mice of susceptible strains develop a demyelinating disease characterized by clinical features of progressive myelopathy, similarly to progressive forms of MS [11], [12]. Since this MS model itself is also based on a viral infection, our study can also be viewed as a bi-pathogenic infection model. Based on the clinical observations reported by Zivadinov et al [8] our hypothesis was that in TMEV infected SJL/J mice, MCMV co-infection will favorably modify the disease course similar to findings observed clinically. Our main outcome measures were disability as assessed by monthly rotarod performance, brain infiltrating lymphocyte analysis by flow cytometry, and MRI based brain atrophy measurements. All of these measures demonstrated a potential protective effect of underlying MCMV infection in this MS model.

## Results

### 1. Preservation of motor function in chronic TMEV infected animals pre-infected with MCMV

**Figure 1:**
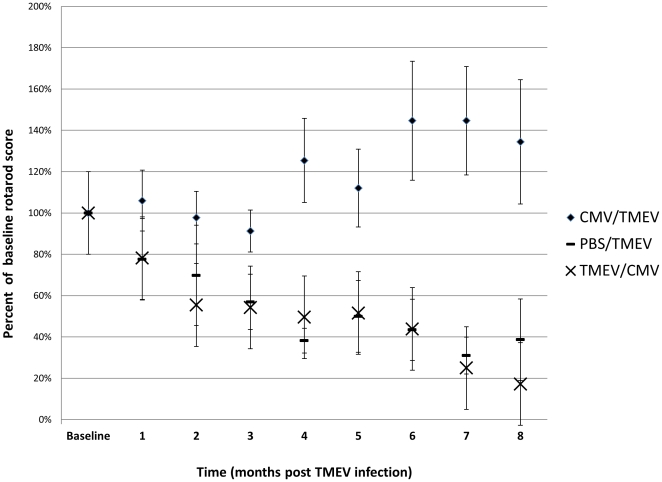
MCMV infection preserves motor function in SJL/J mice undergoing TMEV induced demyelinating syndrome. SJL/J mice infected with MCMV two weeks prior to TMEV infection have significantly higher rotarod scores compared to controls that received PBS injection instead of MCMV The intergroup difference first reaches significance at 3 months, and remains significant beyond that time point (p≤0.024). The figure also shows data acquired in mice infected with MCMV two weeks after TMEV infection; the data in those mice is virtually identical to our PBS/TMEV control group. Error bars represent SD.

To determine the effect of underlying MCMV infection in the TMEV model of MS, a total of four groups of mice were compared. One group of mice received MCMV i.p. 2 weeks prior to i.c. infection with TMEV. The other group received i.p. PBS injection as a control instead of MCMV. We chose to infect the MCMV-infected mice with TMEV at 2 weeks after MCMV infection to allow sufficient time for a MCMV-specific immune response to be developed and for multiple tissue sites to be infected with MCMV [13]. Two additional groups received TMEV infection first, followed by MCMV infection or PBS. As shown in Figure 1, there was a significant (p≤0.024) protective effect of MCMV pre-infection (MCMV/TMEV group) from the standpoint of rotarod detectable functional disability, which first became significant at 3 months post TMEV infection and persisted beyond that. We also noticed the effects of ongoing motor learning resulting in better than baseline performance in these mice; however, this was not statistically significant (p≥0.34). A similar protective effect was not seen in the TMEV/MCMV group, where MCMV infection was 2 weeks after TMEV infection (p≥0.44). Of note, MCMV infection appeared to be controlled at 8 months after infection since in the mice co-infected with TMEV as MCMV replication in the salivary glands, a site for persistent replicating virus, was not detected utilizing the MCMV assay as described under “methods”. In addition, no adverse effects, such as weight loss and inactivity, were observed during the acute infection phase for both the co-infected mice.

### 2. MRI results related to the development of brain atrophy

**Figure 2:**
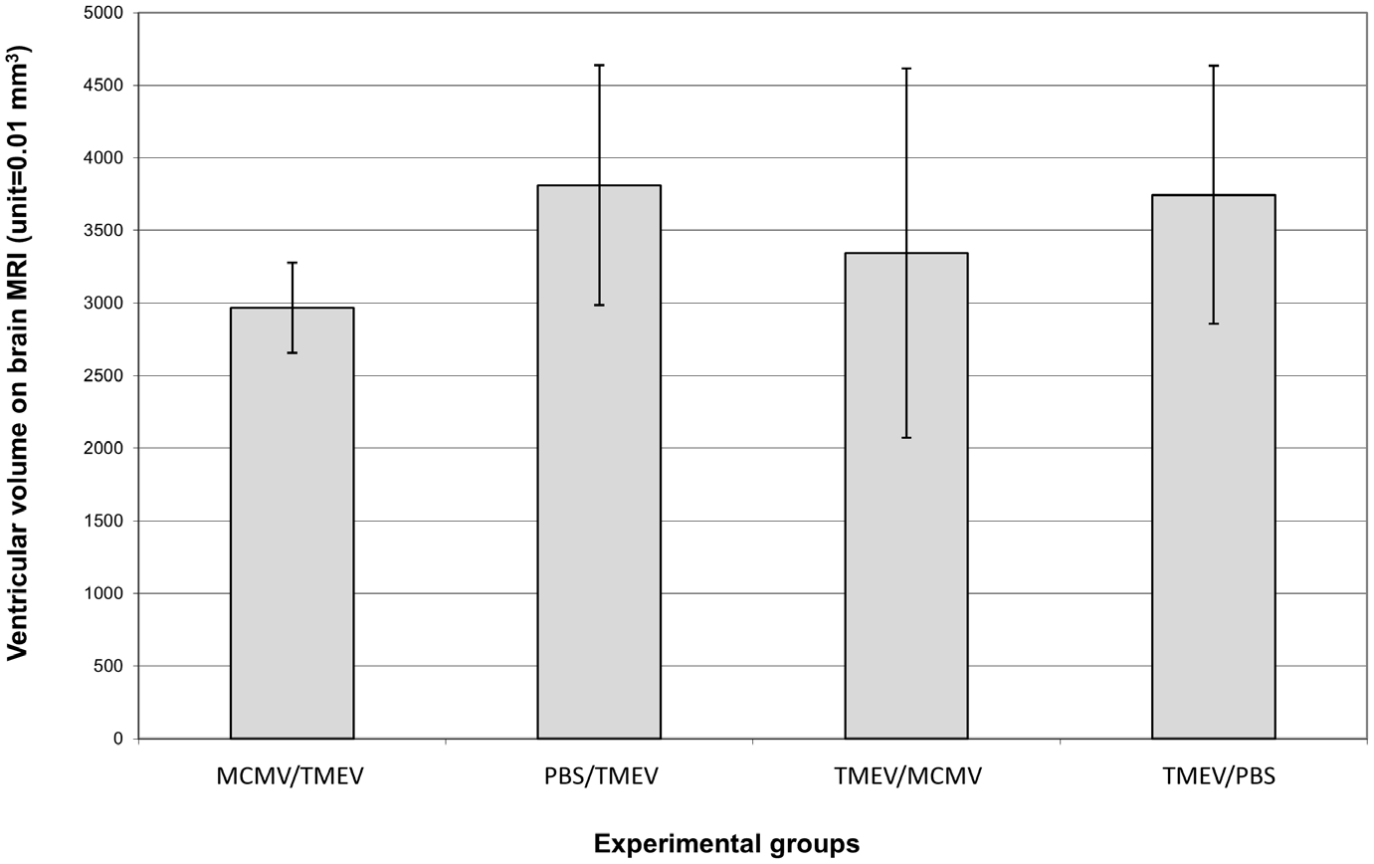
MRI results. Ventricular volumetry at 8 months post TMEV infection. Lower numbers represent less atrophy. The observed close to 30% lower atrophy in the MCMEV/TMEV group only showed a trend (p = 0.19) due to low statistical power, which was the consequence of the relatively high standard deviation observed in these groups. Of note, ventricular volumetry of SJL/J mice undergoing normal aging does not demonstrate the development of significant atrophy as demonstrated earlier [15].

We analyzed brain atrophy in the treated mice. As reported by us previously, brain atrophy is a standard feature of the TMEV infected SJL/J mice [14]. Age-related brain atrophy in this strain is minimal and did not reach statistical significance in our published experiments [14]. We therefore elected to determine the extent underlying MCMV infection reduced brain atrophy in TMEV infected animals. Measuring ventricular volumes, we observed a close to 30% reduction in brain atrophy in TMEV infected animals with underlying MCMV infection (Figure 2); however, while suggestive of a trend towards a protective effect, this experiment was underpowered to detect a statistically significant difference (p = 0.19). The main reason for this was the relatively high standard deviation of ventricular volume in the studied groups of mice. Based on the observed standard deviation, we would have needed 14 mice per group to demonstrate a significant difference. It is important to note that the normal aging of SJL/J mice doesn't include the development of significant atrophy, as demonstrated earlier [15]. In addition, brain MRI metrics other than atrophy were not considered in this model, as the majority of demyelinating lesions are located in the spinal cord and not in the brain. In vivo spinal cord imaging was not considered due to the limited resources available for our study.

### 3. Brain infiltrating lymphocyte analysis by FACS

**Figure 3:**
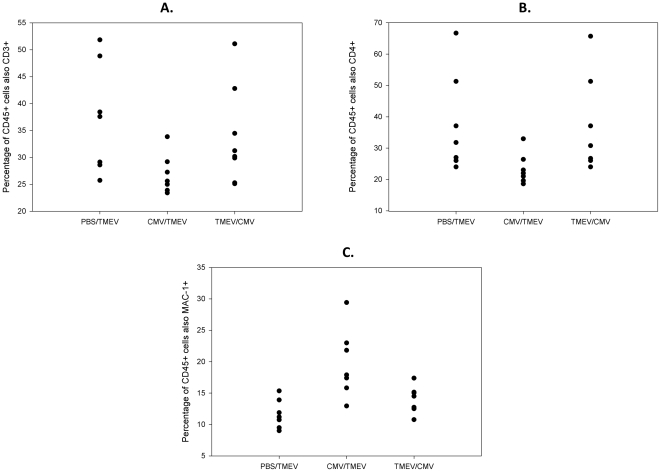
Flow cytometric analysis of brain infiltrating immune cells in 8 months TMEV infected mice that were either sham/PBS injected, pre- or post-infected with MCMV. Shown are CD45+ cells that are: A.) CD3+, B.) CD4+, and C.) Mac1+, respectively. The observed reduction in CD3+ cells compared to PBS controls was significant (p = 0.026), and so was the increase in MAC1+ cells (p = 0.003). The decrease in CD4+ T-cells demonstrated a trend (p = 0.17).

To determine whether the preserved motor ability observed in mice infected with MCMV prior to TMEV infection was due to altered brain infiltrating lymphocytes, we collected and quantified the expansion of CD45+ brain infiltrating immune cells at 8 months post infection. As shown in Figure 3, MCMV pre-infection of TMEV infected mice resulted in a significant reduction reduced numbers of CD3+ cells as a percentage of brain infiltrating CD45+ cells in the brain (p = 0.026). As part of this decrease in CD3+ cell proportion, CD4+ cells exhibited a trend towards reduction in TMEV infected mice pre-infected with MCMV (p = 0.17). Meanwhile, the proportion of CD45+ Mac1+ cells significantly increased in these animals (p = 0.003, Figure 3). Finally, the proportions of B220+ or CD8+ cells did not change significantly in TMEV infected animals (data not shown) pre-infected with MCMV. In contrast, we did not observe statistically significant changes in the proportions of CD3, CD4, CD8, Mac-1 or B220 positive cells infiltrating the brains of TMEV infected mice that were subsequently infected with MCMV 2 weeks later. Overall, these data suggest that pre-infection with MCMV reduces the proportion of CD3+ T cell infiltration in the brain of mice subsequently infected with TMEV. In the same animals, underlying MCMV infection increases the proportion of Mac-1+ macrophage infiltration in chronic TMEV infected animals.

## Discussion

Overall, our findings imply a beneficial immunomodulatory effect of MCMV infection in the TMEV infection MS model. MCMV prior to TMEV infection may therefore contribute to our understanding to the clinical observation that underlying CMV infection contributes to protection from MS. The overall reduction in the proportion of CD3+ cells and the observed increase in the proportion of Mac-1+ macrophages may contribute to this effect.

The role and significance of HCMV infection from the standpoint of MS development remains controversial. Similarly to most putative viral causes of MS, one key issue is the almost ubiquitous positivity of the average population to markers of these infections. A potential causative role for HCMV was suggested over 30 years ago based on primate experiments in which a strain of CMV was isolated from the brain and lymph nodes of a chimpanzee that developed paralysis after intracerebral inoculation with brain cell cultures derived from an MS case [7]. However, most studies have been unable to confirm this purported HCMV association with MS. Meanwhile, a potential disease initiating role for EBV was suggested as far back as 1983 in a study where the HCMV infection rate and HCMV complement fixing antibody production was found to be lower in MS cases [16]. However, a study on post-mortem brain tissue using PCR based detection techniques failed to find any statistically significant association between MS and common viral infections including HCMV and EBV [17]. Meanwhile, a study in Norway found elevated titers to EBV but not to HCMV in MS cases [18]. Recently, Ascherio reported a pathogenic role of EBV in MS whereas no similar association was found regarding HCMV [5]. More recently, EBV-specific CD8+ T-cell responses were shown to be decreased in patients with clinically isolated syndrome (CIS). In contrast, there was no difference between categories for EBV-specific CD4+ T cell, or for HCMV-specific CD4+ and CD8+ T-cell responses [19]. Intrathecal enrichment in EBV-, but not HCMV-specific CD8+ CTL was also reported in early MS patients by another group [20]. EBV but not HCMV IgG antibody indexes were also increased in the CSF in this study. These studies overwhelmingly demonstrate that while EBV may be an important “trigger” of MS development, HCMV doesn't contribute to MS pathogenesis.

A surprising finding about HCMV was its potential protective role in MS, both from the standpoint of MRI and functional outcome measures, suggesting that HCMV infection in MS patients results in a beneficial modulation of the immune response [8]. This previous study demonstrated that recent HCMV infection either by 1) primary infection or 2) secondary infection (since humans can be infected with multiple CMV strains), or 3) reactivation of latent virus leading to recent replication has occurred in those patients, as reflected by the increased HCMV antibody titers. Therefore, it is possible CMV-specific T cells were activated or cytokine induction occurred, which has had an immunomodulatory effect resulting in attenuated MS phenotype. It is clear that both HCMV and MCMV infection affect the responsiveness of T cells since CMV infected dendritic cells can modulate naive and antigen specific T cell responses [21]. There is also evidence that HCMV may cause activation of immunomodulating and immune evasion mechanisms [22], [23] which could alter the adaptive immune processes involved in the pathogenesis of MS [24]. The role of HCMV as an immunomodulating virus has been recognized in solid organ transplantation where it appears to contribute to the immunosuppressed state [25].

Infection of mice with MCMV reflects the infection of humans with HCMV in many respects. MCMV infects numerous tissues and cell types during the acute phase of infection and establishes a lifelong persistent or latent infection similar to HCMV [13]. Similar to HCMV, MCMV encodes viral genes which function to evade or alter the host immune response [9], [10]. Some of these appear to contribute to the observed lifelong viral persistence, while others exploit immune cells that contribute to antiviral immunity [24]. Both HCMV and MCMV inhibit MHC class I expression on infected cells [9], [24], [26], and MCMV has been shown to impair IFN-gamma induced MHC class II-dependent antigen presentation by macrophages [27], [28]. HCMV was also demonstrated to inhibit the induction of HLA class II antigens by IFN-beta dependent and independent molecules including ICAM-1 and VCAM-1, which are thought to be involved in MS pathogenesis [29]. In addition, CMV-infected endothelial cells have the capability to induce IFN-beta production [30]. Interferon beta represents the most commonly used disease modifying agent for relapsing forms of MS [1]. Another possible mechanism to explain the effects of prior MCMV infection on TMEV-mediated MS development could result from modulation of the immune response to TMEV infection itself. In mouse infection studies with either MCMV or other viruses, prior infection of mice with one virus influences the immune response to other heterologous infections [31], [32], [33]. Thus, prior MCMV infection in the TMEV model of MS could lead to attenuation of the MS-like disease as shown in our studies by modulation of the immune response to TMEV infection. In addition, cytokines released as part of an antiviral immune response can activate the hypothalamo-hypophyseal axis, resulting adrenal glucocorticoid release, which in turn provides strong negative feedback on the further synthesis and release of cytokines, and exerts an overall protective effect from the detrimental consequences of an overactive immune response [34], [35]. Lastly, as another potential, but at this stage purely speculative explanation to our observations of increased proportion of Mac-1+ cells, these cells may contribute to a more efficient elimination of TMEV infected CNS cells, and as such led to a better overall outcome. In addition, macrophages may exert immunosuppressive effects on T-cells, as commonly demonstrated in cancer models.[36], [37]

Given that our study was designed as a pilot project paving the way to future larger scale proposals, there are clear limitations to our data. These include the relatively low number of mice per group, which resulted in being underpowered from the standpoint of demonstrating significant MRI-based differences. In addition, due to the same limitation, only one time point was studied with FACS, and we only studied brain and not spinal cord samples - ideally both should be assessed given the prevalence of TMEV in the spinal cord at late time points. We utilized the most commonly studied immune cell markers in the FACS based studies; however, additional immune subset markers, cytokine assay, microarray studies would enable us to study this phenomenon in more details, and all the above are planned in future extensions of this study. The 2-week lag between MCMV and TMEV infections was chosen because MCMV virus titers peak in the salivary gland at 2 weeks and all other tissues are infected and some are even starting to be cleared from the tissue, indicating virus-specific immune response. However, it is possible that a different lag time may have resulted in enhanced immunomodulatory effects. We also did not demonstrate the effects of MCMV alone on the observed outcome measures, including there was no control group where SJL/J mice would be infected with MCMV alone. Histology time course analysis was also not done, but given that we clearly documented significant differences in motor performance, and motor performance in this model is determined by the extent of both demyelination and axonal loss, we anticipate that quantitative measures of the aforementioned would also have demonstrated significant differences.

In conclusion, in the studied chronic-progressive model of MS, MCMV infection prior to demyelinating disease induction resulted in an attenuated clinical phenotype. Overall, the mechanisms by which human or mouse CMV effectively results in reduced disease activity in MS or in the studied MS model remain unclear. However, this study recapitulates in a rodent model the clinical observation that underlying HCMV infection is protective in human MS. We plan to study the observed phenomenon in additional details in future extensions to this study, which will address all the limitations of the current study, and include additional time points and outcome measures. In our view, the presented new model system will enable us to gain insights into the beneficial immunomodulatory mechanism(s) associated with this common viral infection, and may pave the way to novel therapeutic strategies in MS.

## Materials and Methods

### Mice and Experimental Infection

The study was approved by the institutional committee for animal care and use at the University of Cincinnati (approval number 06-10-09-01). 4 week old female SJL/J mice were purchased from the Jackson Laboratory (Bar Harbor, Maine). Four groups of 8 mice were studied: MCMV inoculation either preceded the infection with TMEV by 2 weeks, or was established 2 weeks after TMEV. We also used 2 control groups where PBS was used as control for the MCMV inoculation, either 2 weeks before or 2 weeks after infection. To induce the MS-like demyelinating disease, all groups received TMEV infection by intracranial (i.c.) injection of 10^5^ PFU of TMEV from the DAV strain as published earlier [38], [39]. Mice that received MCMV were inoculated by intraperitoneal (i.p.) injection of 1×10^5^ PFU MCMV (K181+, salivary gland passaged stock [40]). Matching control groups received PBS instead of MCMV (PBS/TMEV and TMEV/PBS groups). All mice were followed for a total of 8 months after TMEV infection. Our earlier observations suggested that mice reach their peak disability by approximately 240 days post infection [14]. Premature animal loss due to co-infection was not observed during this study.

### Viral assays

At the end of the study, tissues were collected to determine the effects of co-infection on normal levels of MCMV or TMEV infection. At 8 months post infection, salivary glands were collected to determine whether MCMV continued to persist or replicate as a result of the TMEV co-infection. As previously described, 10% salivary gland tissue homogenates (w/v) were sonicated and plaque assays performed on NIH 3T3 cells [41], [42]. Following incubation of the monolayers for 6 days under carboxymethylcellulose-2X media, the cells were stained with Giemsa and plaques enumerated by light microscopy. The limit of detection for the plaque assay is <10 PFU/ml of tissue homogenate.

### Disability measurement

The rotarod assay (Rotamex rotarod, Columbus Instruments, Columbus, OH) was performed as a functional outcome measure (assessment of disability) every month, as previously published [38]. Mice were trained on the rotarod daily for one week prior to infection to minimize effects of motor learning.

### Brain atrophy measurements

MRI based brain volumetry was performed to assess brain atrophy at the last time point (8 months post TMEV). For image acquisition, a Bruker Biospec 7 Tesla horizontal bore small rodent MR imaging system was used as described earlier [14]. A T2 weighted three dimensional RARE sequence was utilized for data acquisition (TR: 1500 ms, effective TE: 65 ms, RARE factor: 16, isometric 125 micron resolution, total acquisition time ∼40 minutes). We analyzed atrophy by performing volumetric measurements of the ventricular enlargement as reported earlier [14]. Briefly, volumetric analysis using the 3D ROI tool was conducted using the Analyze software package, developed by Mayo Clinic's Biomedical Imaging Resource [43], [44].

### FACS analysis

Mice were euthanized and their brains were harvested at 8 months post infection, immediately following the MRI acquisition. Brain-infiltrating lymphocytes were isolated from mouse brain through collagenase digestion and a percoll gradient as previously described [45], [46]. Inflammatory cells isolated from the brains of each mouse were stained with anti-CD4 PE (BD catalog #553730), anti-CD8 PerCP (BD catalog #553036), anti-CD3 APC (BD catalog #553066), anti-CD45 PE-Cy7 (BD catalog #552848), anti-B220 FITC (BD catalog #553087), and anti-Mac-1 FITC (BD catalog #55557396) antibodies. Samples were then washed twice with fluorescence-activated cell sorting buffer, resuspended in cold phosphate-buffered saline, and fixed in 1% paraformaldehyde. Samples were then analyzed on a BD LSRII instrument (BD Biosciences) [47], [48].

### Statistical analysis

Intergroup differences were analyzed statistically using standard statistical methods in JMP! (SAS Institute, Cary, NC) and SigmaPlot (Systat Software, Chicago, IL).
